# Intramedullary fixation versus plate fixation in the treatment of midshaft clavicle fractures: a meta-analysis of randomized controlled trials

**DOI:** 10.3389/fsurg.2023.1194050

**Published:** 2024-07-11

**Authors:** Minpeng Lu, Hao Qiu, Yuting Liu, Jing Dong, Lingfang Jiang

**Affiliations:** ^1^ Department of Pain Medicine, The First Affiliated Hospital of Chongqing Medical University, Chongqing, China; ^2^ Department of Orthopedics, The Second Affiliated Hospital of Chongqing Medical University, Chongqing, China; ^3^ Department of Endocrinology, The Ninth People’s Hospital of Chongqing, Chongqing, China; ^4^ Department of Clinical Medicine, Chongqing Medical and Pharmaceutical College, Chongqing, China; ^5^ Experimental Teaching Management Center of Chongqing Medical University, Chongqing, China

**Keywords:** clavicle, intramedullary fixation, midshaft clavicle fractures, meta-analysis, plate fixation

## Abstract

**Objective:**

The aim of this systematic review and meta-analysis is to assess the clinical efficacy of intramedullary fixation (IF) vs. plate fixation (PF) in the treatment of midshaft clavicle fractures.

**Methods:**

We conducted a computerized search of the electronic databases (PubMed, EMBASE, Cochrane Library, Medlineand Chinese Journal Full-text Database) from the establishment of the database to the end of November 2022. The quality of the included studies was assessed according to the Cochrane Collaboration's “Risk of bias”. Comparisons between the two groups were based on 8 variables, including Constant score, disabilities of the arm, shoulder and hand (DASH) score, surgery time, length of incision, hospital stay; time to union, blood loss and infection.

**Results:**

Thirteen randomized controlled trials (RCTs) comprising a total of 928 patients were included in our meta-analysis. The pooled results showed that IF can benefit midshaft clavicle fractures with a reduced surgery time and hospital stay, a smaller incision, a better shoulder function (DASH score), shorter time to union and lower rate of infection compared with PF. However, there was no significant difference between the two groups in terms of Constant score at 12-month follow-up.

**Conclusion:**

IF is superior to PF for the treatment of midshaft clavicle fractures.

## Introduction

Fractures of the clavicle are common injuries, constituting 2.6%–5% of all fractures in adults (1). Approximately 80% of such fractures are midshaft fractures, and over half of the latter are displaced (2–4). Historically, midshaft clavicle fractures were treated conservatively, with a sling or a figure-of-eight bandage, because they were thought to heal with high rates of union and good patient satisfaction (5–10). Recent studies, however, have emphasized that the risk of nonunion following conservative treatment is higher than previously reported (11–13). Therefore there has been a shift toward the operative treatment of midshaft clavicle fractures.

Intramedullary fixation (IF) and plate fixation (PF) are two of the most commonly used surgical treatments for midshaft clavicle fractures (14). Although PF has been a preferred method owing to high union rates and good functionality, it also has drawbacks, such as higher rates of infection, skin irritation from implant prominence, implant failure and refracture after implant removal (15). IF has the advantages of preventing plate irritation, thus decreasing the incidence of infection, preserving the soft tissue envelope and periosteum, and reducing the length of incision (16, 17). Potential limitations involve high rates of nonunion and painful prominent hardware.

Although some published systematic reviews and meta-analyses have assessed the clinical effects of IF vs. PF in the treatment of midshaft clavicle fractures (18, 19), they did not analyze all the outcome indicators because they lacked the relevant data. Now, because several relevant newrandomized controlled trials (RCTs) have been published, we have been able to provide more comprehensive, convincing, and useful information in our updated meta-analysis.

## Materials and methods

### Search strategy

We conducted a computerized search of several electronic databases (e.g., PubMed, EMBASE, Cochrane Library, Medline and Medlineand Chinese Journal Full-text Database) from the establishment of the database to the end of November 2022, according to the Preferred Reporting Items for Systematic Reviews and Meta-Analyses (PRISMA) for published studies comparing IF with PF in patients with midshaft clavicular fractures. The key words used included *midshaft clavicle fractures, clavicle fractures, plate, intramedullary, randomized controlled trials, and randomized*. Secondary searches of unpublished literature were conducted by searching the Google Scholar and Medical Matrix up to the end of November 2022. The references in these articles were also searched to identify any additional studies not previously identified in the initial literature search.

### Eligibility criteria

Studies with the following criteria were included: (1) RCTs comparing IF with PF in the treatment of midshaft clavicle fractures; (2) patients between 16 and 70 years of age; (3) interventions including IF (intramedullary pin, Knowles pin, or Rockwood clavicle pin) and/or PF (locking plate or reconstruction plate); (4) at least a 12-month follow-up. The exclusion criteria were as follows: (1) duplicate or multiple publications of the same study, retrospective studies, or case reports; (2) studies reporting only elderly patients (age >60 years); (3) studies not reporting interested outcomes.

### Quality assessment

The quality of each of the included studies was independently assessed by 2 reviewers (according to the Cochrane Collaboration's “Risk of bias” criteria). The Cochrane Risk of Bias Tool of Review Manager version 5.4 (Copenhagen, Denmark: Nordic Cochrane Centre, Cochrane Collaboration) was applied. Appraisal criteria included: random sequence generation, allocation concealment, blinding of participants and personnel, blinding of outcome assessment, incomplete outcome data, selective reporting, and other biases. Each of these factors was recorded as denoting low risk, unclear risk, or high risk. Where data were unclear, we contacted authors, when possible, for clarification. Disagreements were resolved by third party adjudication.

### Data extraction

Two reviewers independently extracted and cross-checked the data. The decision to include studies was made initially on the basis of the study title and abstract. When a study could not be excluded with certainty at this stage, the full-text was obtained for evaluation. Disagreements were resolved by discussion and, where necessary, in consultation with a third reviewer. Extracted information included the first author, publication year, study design, characteristics of participants and information to assess the risk of bias. If any data were missing from the trial reports, the reviewers attempted to obtain the data by contacting the authors.

### Outcome

Primary outcomes were Constant score, DASH score, and time to union. The secondary outcomes were classified as surgery time, length of incision, hospital stay, blood loss and infection.

### Statistical analysis

The statistical analysis was performed with RevMan 5.4. For dichotomous variables, the risk ratio (RR) with a 95% confidence interval (CI) was calculated. For continuous variables, mean difference (MDs) with a 95% CI or standardized mean difference (SMD) with a 95% CI was calculated. A value of *P* < 0.05 was regarded as statistically significant. The assessment for statistical heterogeneity was calculated using the chi^2^ statistic and *I*^2^ statistic. If there was no heterogeneity (*P* > 0.10, *I*^2^ < 50%), a fixed effect model was used. Otherwise, a random effect model was used. The outcome of meta-analysis for variables was summarized graphically using a forest plot. The presence of publication bias was assessed using Egger tests. Publication bias was observed with the funnel plot. We were unable to perform pooled analyses as results varied widely with exclusion of any study.

## Results

### Search results

**Figure 1:**
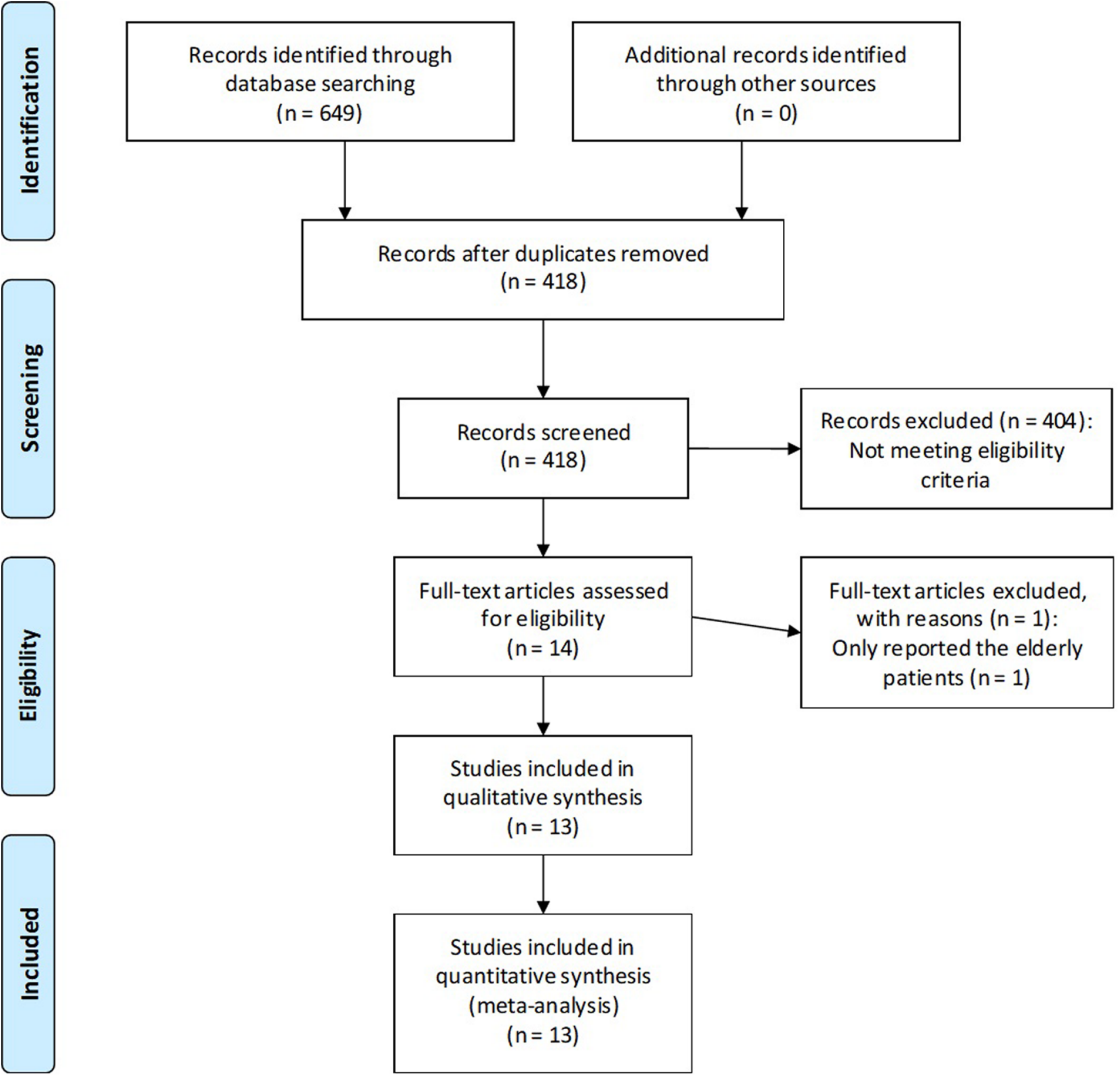
PRISMA 2009 flow diagram.

A total of 649 records were reviewed. After removal of duplicates, 231 records were screened; 404 studies clearly did not match our inclusion criteria by title and abstract and were therefore excluded. A RCT was excluded because it reported only elderly patients (20). Finally, 13 studies met the eligibility criteria and were found suitable for our meta-analysis (21–33). All studies were prospective RCTs. The study selection process and reasons for exclusion are summarized in Figure 1.

### Quality assessment and basic information

**Figure 2:**
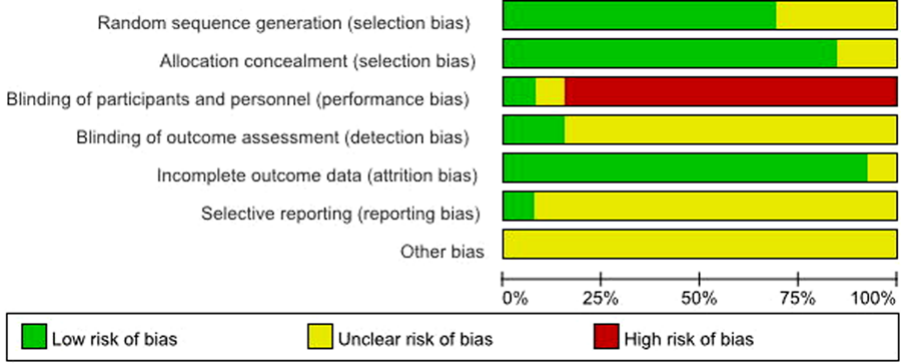
Risk of bias graph.

**Figure 3:**
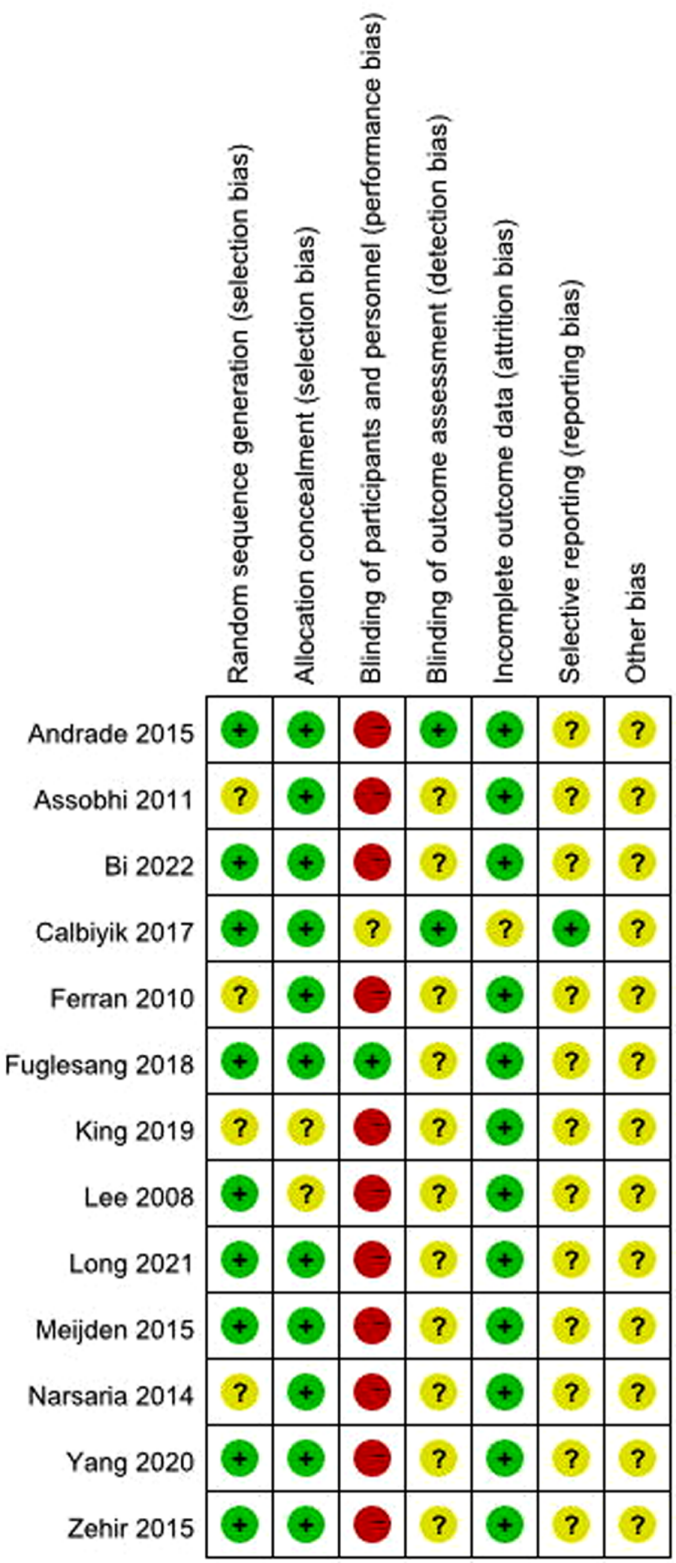
Risk of bias summary.

The quality of the included studies (13 RCTs) was assessed using the Cochrane Collaboration's “Risk of bias” criteria. The risk of bias assessment of included studies is given in Figures 2, 3. Thirteen RCTs published between 2008 and 2022 were included; and a summary of their characteristics is presented in Table 1. A total of 928 patients (477 in the IF group and 451 in the PF group) were enrolled in these studies. As described in each study, patients treated by both methods were comparable in terms of gender, side involved and injury mechanism. All of the studies involved patients with midshaft clavicle fractures who were followed for at least 12 months. All the articles evaluated the clinical efficacy of IF and PF in the treatment of midshaft clavicle fractures. The IF group included intramedullary pins, Knowles pins and Rockwood pins. The PF group included locking plates and reconstruction plates.

**Table 1:** Characteristics of the included studies.

Study	Country	Study type	*n*		Age		Follow-up (months)
			IF (M/F)	PF (M/F)	IF	PF	
Lee et al. (28)	China	RCT	37/19	20/12	40.1	28.2	≥12
Ferran et al. (27)	UK	RCT	14/3	13/2	23.8	35.4	≥12
Assobhi (25)	Egypt	RCT	16/3	17/2	30.3 ± 4.8	32.6 ± 5.9	≥12
Narsaria et al. (29)	India	RCT	24/9	26/6	38.9 ± 9.1	40.2 ± 11.2	≥12
Andrade-Silva et al. (24)	Brazil	RCT	19/7	28/5	28.3 ± 9.4	31.2 ± 12.2	≥12
Meijden et al. (26)	Netherlands	RCT	60/2	53/5	39.6 ± 13.2	38.4 ± 14.6	≥12
Zehir et al. (32)	Turkey	RCT	14/10	12/9	33.17 ± 8.6	32.38 ± 8.41	≥12
Calbiyik et al. (21)	Turkey	RCT	21/14	25/15	42.02 ± 13.87	39.07 ± 7.04	≥12
Fuglesang et al. (22)	Norway	RCT	46/8	52/8	37.4	34.9	≥12
King et al. (23)	South Africa	RCT	26/9	20/17	29 ± 14	35 ± 12	≥12
Yang et al. (30)	China	RCT	37/21	34/21	41.2 ± 12.4	42.6 ± 15.1	≥12
Long et al. (33)	China	RCT	25/5	22/8	36.4 ± 10.3	36.6 ± 10.4	≥12
Bi et al. (31)	China	RCT	15/11	16/10	31.57 ± 5.21	35.09 ± 5.4	≥12

RCT, randomized controlled trial; IF, intramedullary fixation; PF, plate fixation; M, males; F, females.

### Outcomes

#### Primary outcomes

**Figure 4:**
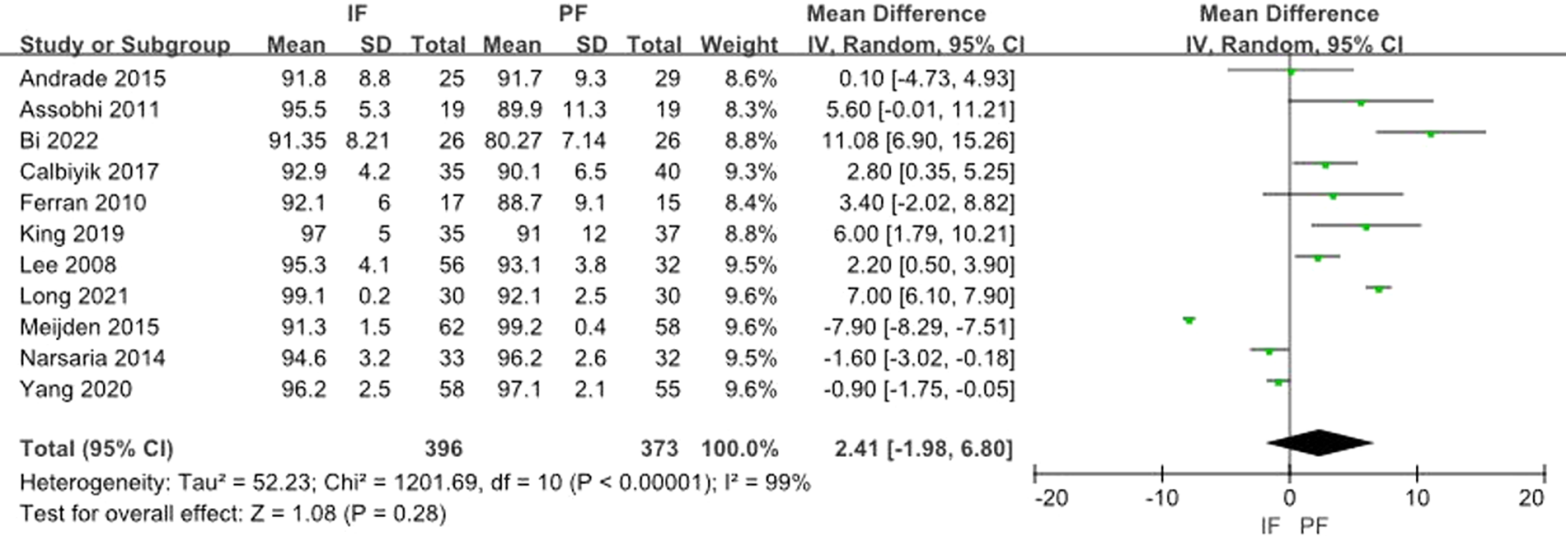
Forest plot of comparison: constant score.

Eleven studies reported the Constant score. The heterogeneity test showed a significant heterogeneity (*I*^2 ^= 99%) and the random effect model was applied. The aggregated results showed that there was no statistical difference between the 2 methods (MD: 2.41, 95% CI: −1.98 to 6.80) (Figure 4).

**Figure 5:**
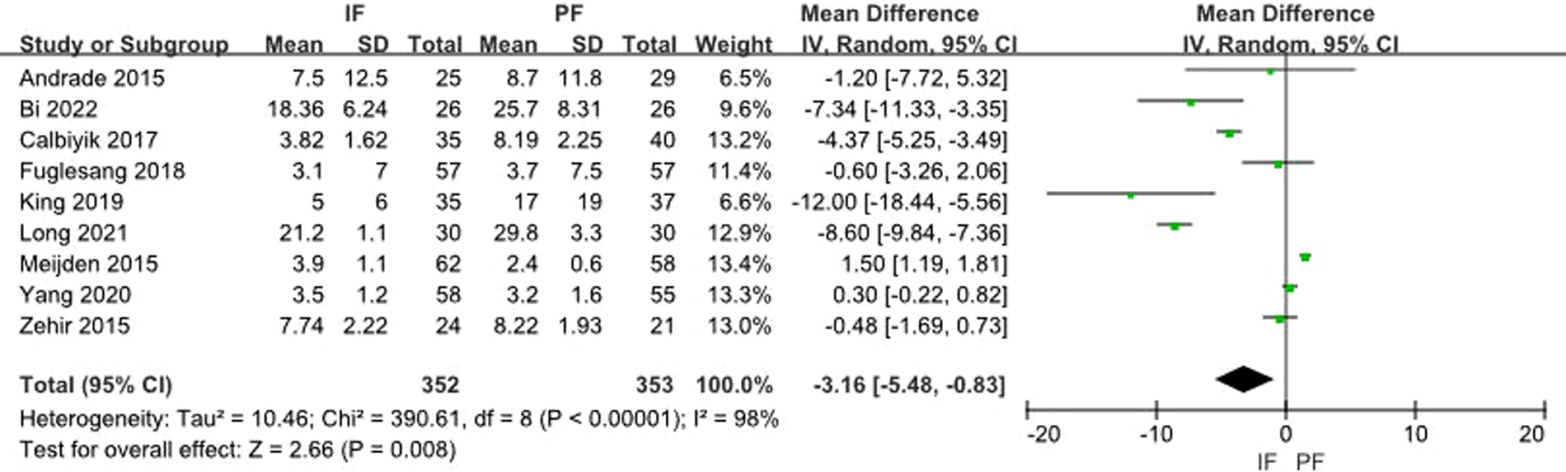
Forest plot of comparison: DASH score.

Nine studies reported the DASH Score. The heterogeneity test showed a significant heterogeneity (*I*^2 ^= 98%) and the random effect model was applied. A meta-analysis of the aggregated results showed a statistical difference between the two groups (MD: −3.16, 95% CI: −5.48 to −0.83) (Figure 5).

**Figure 6:**
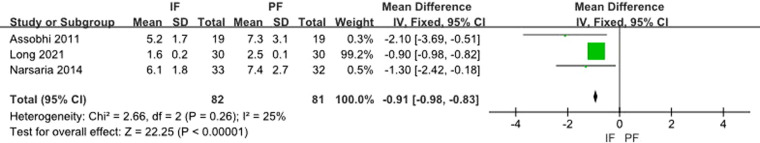
Forest plot of comparison: union time (month).

**Figure 7:**
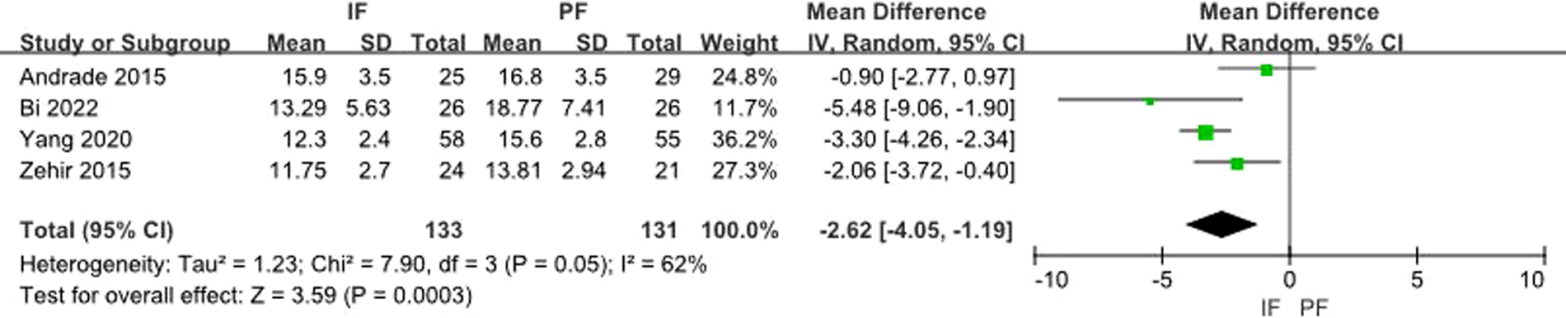
Forest plot of comparison: union time (week).

Time to union was reported by 8 studies; Three (25, 29, 33) of these used month as a unit, and the meta-analysis indicates their significance (MD: −0.91, 95% CI: −0.98 to −0.83) without any heterogeneity (*I*^2 ^= 25%) (Figure 6). 4 (24, 30–32) used the week as a unit, and the meta-analysis indicates their significance (MD: −2.62, 95% CI: −4.05 to −1.19) with heterogeneity (*I*^2 ^= 62%) (Figure 7). One study used the day as a unit, and a significant difference was found in both groups (*P* = 0.26) (28).

#### Secondary outcomes

##### Perioperative data

**Figure 8:**
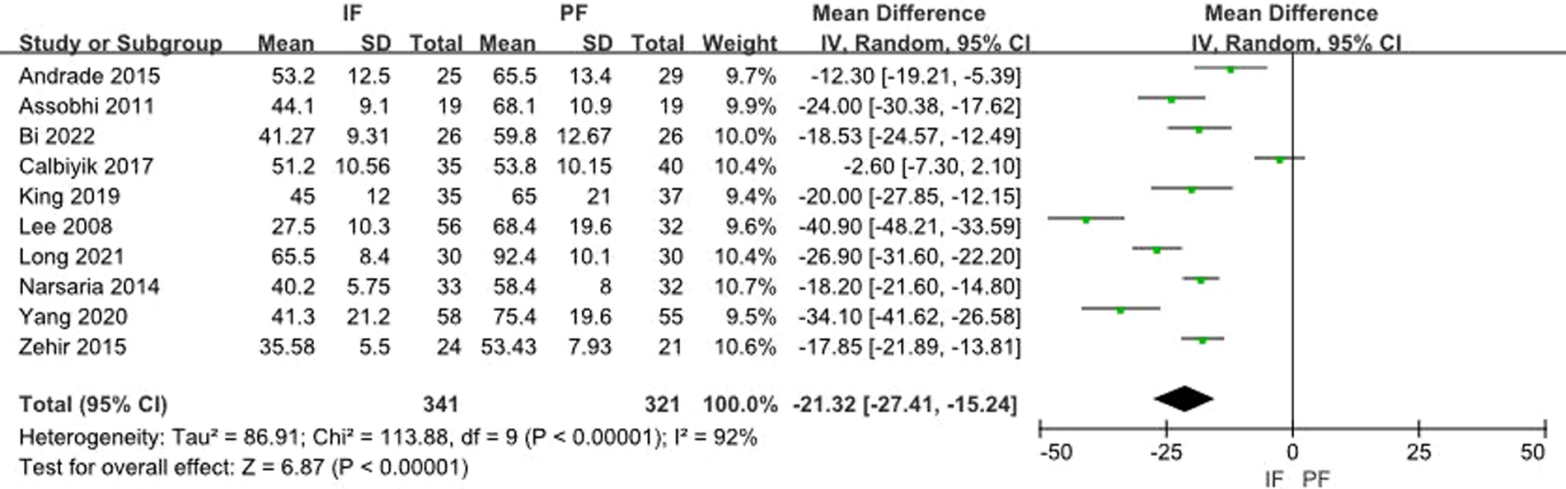
Forest plot of comparison: surgery time.

Ten studies reported the surgery time. The meta-analysis for aggregated results from 10 studies showed a significant difference (MD: −21.32, 95% CI: −27.41 to −15.24) with significant heterogeneity (*I*^2 ^= 92%) (Figure 8).

**Figure 9:**
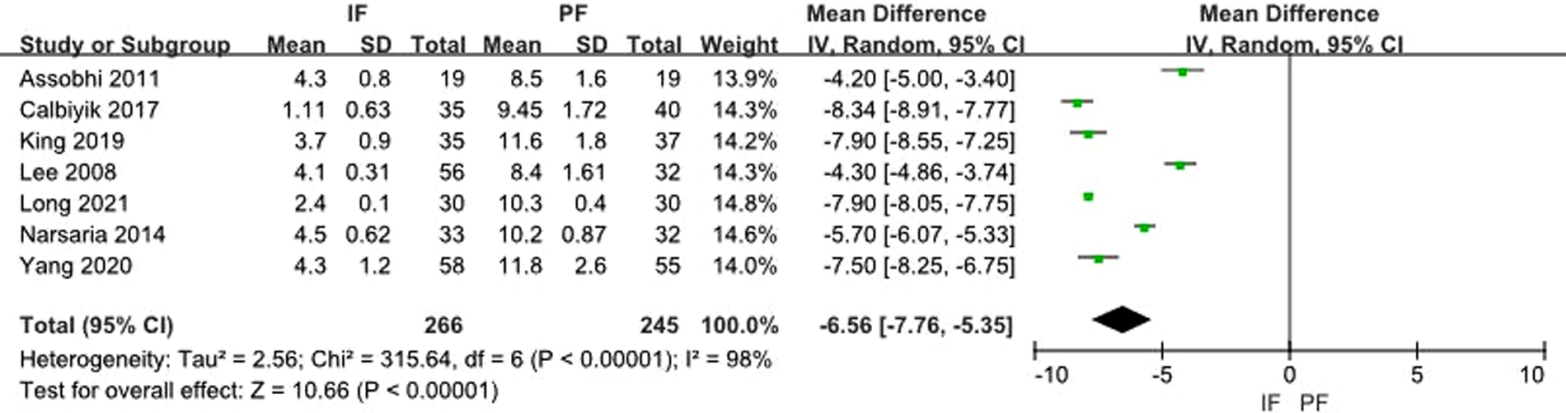
Forest plot of comparison: length of incision.

Seven studies reported the length of incision. The heterogeneity test showed a significant heterogeneity (*I*^2 ^= 98%) and the random effect model was applied. A meta-analysis of the aggregated results showed a statistical difference between the two groups (MD: −6.56, 95% CI: −7.76 to −5.35) (Figure 9).

**Figure 10:**
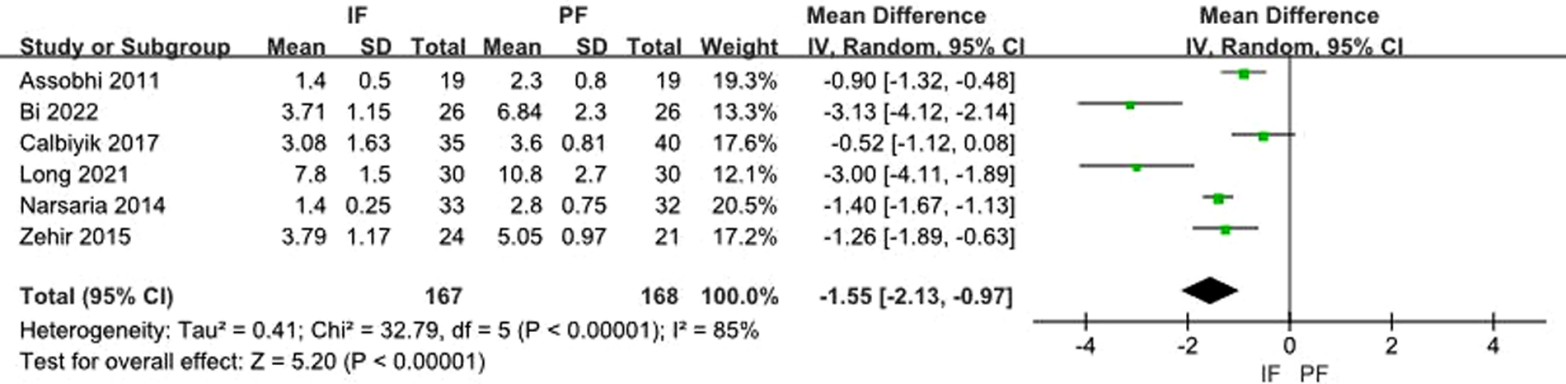
Forest plot of comparison: length of hospital stay.

Six studies reported the length of hospital stay. The heterogeneity test showed a significant heterogeneity (*I*^2 ^= 85%) and the random effect model was applied. A meta-analysis of the aggregated results showed a statistical difference between the two groups (MD: −1.55, 95% CI: −2.13 to −0.97) (Figure 10).

**Figure 11:**
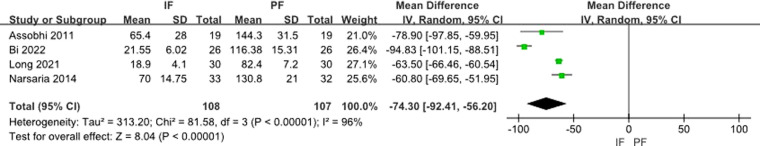
Forest plot of comparison: blood loss.

Four studies reported on blood loss. The heterogeneity test showed a significant heterogeneity (*I*^2 ^= 96%) and the random effect model was applied. A meta-analysis of the aggregated results showed a statistical difference between the two groups (MD: −74.30, 95% CI: −92.41 to −56.20) (Figure 11).

**Figure 12:**
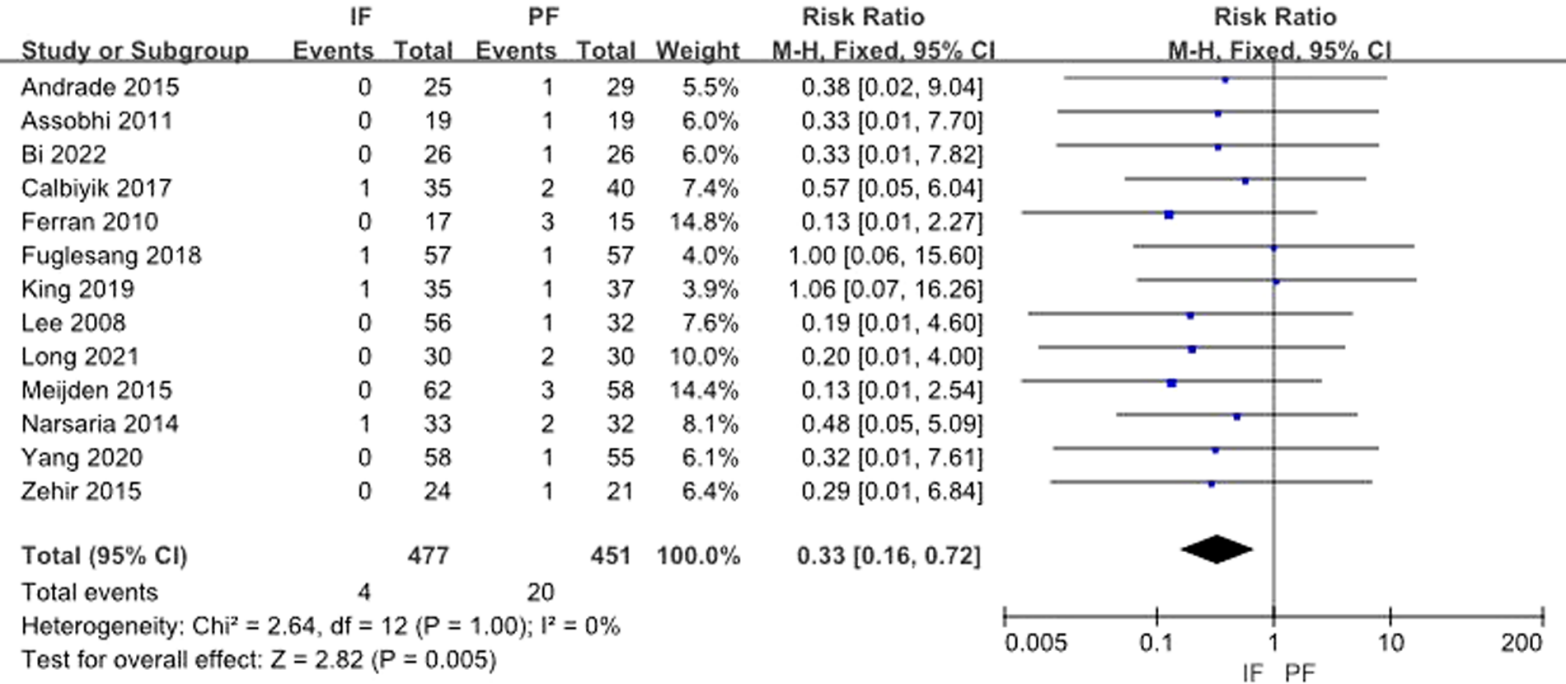
Forest plot of comparison: incidence of infection.

All 13 studies provided data on incidence of infection. The test for heterogeneity was not significant (*I*^2 ^= 0%) and the fixed effect model was adopted. Pooled data showed that a statistical difference existed between the 2 groups (RR: 0.33, 95% CI: 0.16–0.72) (Figure 12).

##### Publication bias

**Figure 13:**
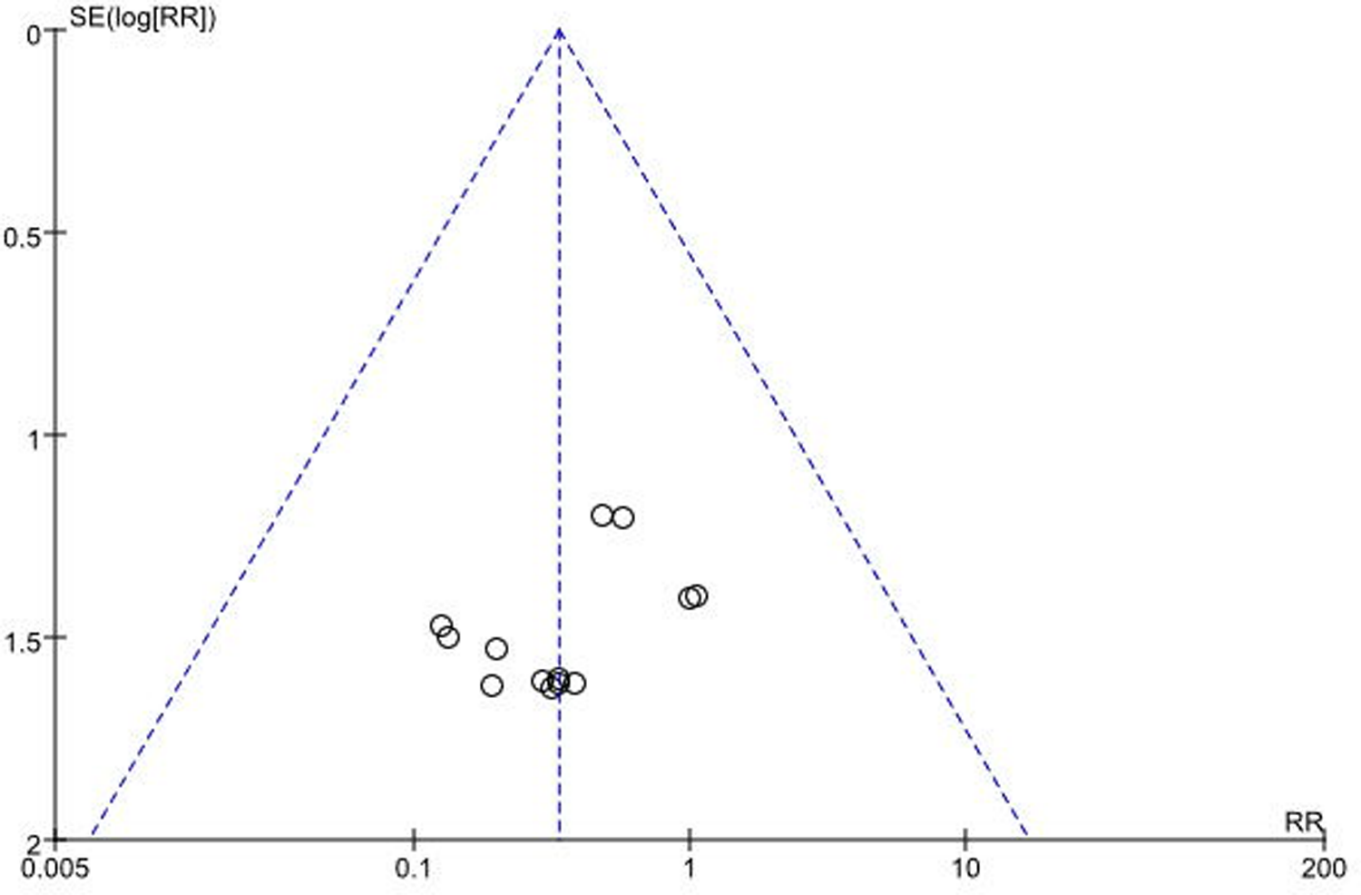
Funnel plot of publication bias.

The funnel plot was symmetrical in general, indicating small publication bias (Figure 13).

## Discussion

The optimal surgical method for midshaft clavicle fractures remains a matter of debate. In the past, PF was most commonly used for fixing midshaft clavicle fractures (34). However, IF is considered as a better selection for midshaft clavicle fractures, and it is associated with a lower incidence of complications (17). In theory, IF and PF both have advantages and disadvantages. Therefore, to provide strong support for clinical decision making, we conducted an updated meta-analysis to determine the optimal surgical method for midshaft clavicle fractures.

The outcomes investigated comprised both primary outcomes (including Constant score, DASH score, and union time) and secondary outcomes (including surgery time, length of incision, and hospital stay; blood loss, and infection). The aggregated results from our meta-analysis of seven RCTs suggest that IF can be of benefit in the treatment of midshaft clavicle fractures with reduced surgery time and hospital stay, a smaller incision, less bleeding, shorter union time and lower rate of infection in comparison with PF. This result was also supported by some previous systematic reviews (35, 36) and meta-analyses (37–39). Furthermore, our meta-analysis indicates that there was no significant difference between the two methods in terms of Constant score and DASH score at 12-month follow-up. Based on current evidence, we conclude that IF is the optimum choice in the treatment of midshaft clavicle fractures.

The meta-analysis with five RCTs by Zhu et al. (39) suggested that IF has advantages over PF with a greater Constant score at 12-month follow-up. But in 2 recent randomized controlled trials, Andrade-Silva et al. (24) reported that there was no significant difference between the 2 techniques, with a similar Constant score at 12-month follow-up (*P* = 0.937) and Meijden et al. (26) agreed. In 4 other randomized controlled trials (25, 27–29), similar results were reported. Furthermore, whether at 6 or 12-month follow-up,6 reports (22, 24, 26, 27, 29, 32) revealed that no discrepancy was observed in terms of DASH score, Oxford Shoulder score, or American Shoulder and Elbow Surgeons (ASES) score. Thus, we suggested that IF and PF yielded similar functional outcomes.

In addition to the results already mentioned, 6 studies (25–29, 32) also separately analyzed other variables such as blood loss, skin irritation, hypertrophic scar, and symptomatic hardware event. We did not merge these variables owing to the lack of more relevant data. From 2 studies (25, 28), however, we concluded that PF significantly increased the risk of hypertrophic scar and symptomatic hardware event. Some reports (25, 26, 29, 32) indicated that the incidence of skin irritation was similar between the groups, whereas mean blood loss was significantly lower in the IF group. Regarding the incidence of malunion, a RCT by Meijden et al. (26) reported that there was no significant difference between the IF and PF groups (0/62 vs. 0/58). A meta-analysis with 4 RCTs and 9 retrospective studies by Zhang et al. (38) and 2 retrospective studies by Chen et al. (40) and Wijdicks et al. (41) also support this conclusion. It is worth mentioning that in terms of biomechanics the PF was stiffer than IF in both pure bending and torque loading (42). But, the absolute stability provided by PF is not an advantage for fracture healing. It is not surprising that PF requires more time for union than IF owing to its requirement for absolute stability. The pooled results by this meta-analysis and Zhu's (39) meta-analysis all showed no significant difference between the 2 groups in terms of refracture after implant removal, whereas the meta-analysis by Zhang et al. (38) determined that IF is associated with a lower incidence of refracture after implant removal. The reason of these conflicting conclusions may be explained by the fact that Zhang's (38) meta-analysis contains some retrospective; case-control studies. Still, the results of our meta-analysis may be not be conclusive owing to the small size of our sample. The present debate about the incidence of refracture after implant removal may be solved in the course of further RCTs.

The present meta-analysis has other potential limitations. First, we do not compare the specific fixators, which may affect the stability and reliability of the conclusions. For instance, IF includes intramedullary pins, Knowles pins, and Rockwood pins. PF also includes locking plate and reconstruction plates. Second, different studies use different inclusion and exclusion criteria and follow-up times, which can create some of the heterogeneity we observed among trials. Third, some indicators appeared in only a single study; more RCTs that focus on these indicators are expected in the future, and these may make our results more convincing. Fourth, although we included only RCTs, the quality of the recruited studies was inconsistent. Some studies had adequate randomization, but others had only incomplete random sequence generation, weak blinding, or imperfect allocation concealment. After enough high-quality studies become available, this limitation may be solved by an updated meta-analysis containing only them.

## Conclusion

In comparing IF and PF in the treatment of midshaft clavicle fractures, the available evidence from our meta-analysis suggests that IF can be of benefit in the treatment of midshaft clavicle fractures owing to reduced surgery time and hospital stay, a smaller incision, shorter time to union, and lower rate of superficial infection in comparison with PF. Although IF and PF yielded similar functional outcomes, PF significantly increased the risk of blood loss, hypertrophic scar, and symptomatic hardware event. Therefore, we conclude that IF is superior to PF for the treatment of midshaft clavicle fractures.

## Data Availability

The raw data supporting the conclusions of this article will be made available by the authors, without undue reservation.

## References

[1] Frima H van Heijl M Michelitsch C van der Meijden O Beeres FJP Houwert RM Clavicle fractures in adults; current concepts. Eur J Trauma Emerg Surg. (2020) 46(3):519–29. 10.1007/s00068-019-01122-4 30944950

[2] Zou R Wu M Guan J. Clavicle shaft fracture after surgery for bipolar dislocation of the clavicle. Am J Case Rep. (2020) 21:e924889. 10.12659/AJCR.924889 33044949 PMC7568524

[3] Ramponi DR Jo Cerepani M. Clavicle fractures. Adv Emerg Nurs J. (2021) 43(2):123–7. 10.1097/TME.0000000000000347 33915562

[4] Chen X Shannon SF Torchia M Schoch B. Radiographic outcomes of single versus dual plate fixation of acute mid-shaft clavicle fractures. Arch Orthop Trauma Surg. (2017) 137(6):749–54. 10.1007/s00402-017-2676-0 28374093

[5] Robinson CM. Fractures of the clavicle in the adult. Epidemiology and classification. J Bone Joint Surg Br. (1998) 80(3):476–84. 10.1302/0301-620x.80b3.8079 9619941

[6] Wijdicks FJ Houwert RM Dijkgraaf MG De Lange DH Meylaerts SA Verhofstad MH Rationale and design of the plate or pin (POP) study for dislocated midshaft clavicular fractures: study protocol for a randomised controlled trial. Trials. (2011) 12:177. 10.1186/1745-6215-12-177 21762476 PMC3160903

[7] Postacchini R Gumina S Farsetti P Postacchini F. Long-term results of conservative management of midshaft clavicle fracture. Int Orthop. (2010) 34(5):731–6. 10.1007/s00264-009-0850-x 19669643 PMC2903171

[8] Biz C Pozzuoli A Belluzzi E Scucchiari D Bragazzi NL Rossin A An institutional standardised protocol for the treatment of acute displaced midshaft clavicle fractures (ADMCFs): conservative or surgical management for active patients? Healthcare. (2023) 11(13):1883. 10.3390/healthcare11131883 37444717 PMC10341159

[9] Biz C Scucchiari D Pozzuoli A Belluzzi E Bragazzi NL Berizzi A Management of displaced midshaft clavicle fractures with figure-of-eight bandage: the impact of residual shortening on shoulder function. J Pers Med. (2022) 12(5):759. 10.3390/jpm12050759 35629181 PMC9145303

[10] Tagliapietra J Belluzzi E Biz C Angelini A Fantoni I Scioni M Midshaft clavicle fractures treated nonoperatively using figure-of-eight bandage: are fracture type, shortening, and displacement radiographic predictors of failure? Diagnostics (Basel). (2020) 10(10):788. 10.3390/diagnostics10100788 33027989 PMC7599597

[11] Kim DW Kim DH Kim BS Cho CH. Current concepts for classification and treatment of distal clavicle fractures. Clin Orthop Surg. (2020) 12(2):135–44. 10.4055/cios20010 32489533 PMC7237254

[12] Moverley R Little N Gulihar A Singh B. Current concepts in the management of clavicle fractures. J Clin Orthop Trauma. (2020) 11(Suppl 1):S25–30. 10.1016/j.jcot.2019.07.016 31992912 PMC6978197

[13] Vaishya R Vijay V Khanna V. Outcome of distal end clavicle fractures treated with locking plates. Chin J Traumatol. (2017) 20(1):45–8. 10.1016/j.cjtee.2016.05.003 28233729 PMC5343094

[14] Smekal V Oberladstaetter J Struve P Krappinger D. Shaft fractures of the clavicle: current concepts. Arch Orthop Trauma Surg. (2009) 129(6):807–15. 10.1007/s00402-008-0775-7 18989685

[15] King PR Lamberts RP. Management of clavicle shaft fractures with intramedullary devices: a narrative review. Expert Rev Med Devices. (2020) 17(8):807–15. 10.1080/17434440.2020.1793668 32635794

[16] Sidhu N Huntington LS Richardson M Ackland DC. Biomechanical performance of an intramedullary Echidna pin for fixation of comminuted mid-shaft clavicle fractures. ANZ J Surg. (2019) 89(10):1308–13. 10.1111/ans.15392 31480097

[17] Wang YC Fu YC Chou SH Liu PC Tien YC Lu CC. Titanium elastic nail versus plate fixation of displaced midshaft clavicle fractures: a retrospective comparison study. Kaohsiung J Med Sci. (2015) 31(9):473–9. 10.1016/j.kjms.2015.07.008 26362960 PMC11915977

[18] Li L Yang X Xing F Jiang J Tang X. Plate fixation versus intramedullary nail or knowles pin fixation for displaced midshaft clavicle fractures: a meta-analysis of randomized controlled trials. Medicine (Baltimore). (2020) 99(39):e22284. 10.1097/MD.0000000000022284 32991430 PMC7523859

[19] Houwert RM Smeeing DP Ahmed Ali U Hietbrink F Kruyt MC van der Meijden OA. Plate fixation or intramedullary fixation for midshaft clavicle fractures: a systematic review and meta-analysis of randomized controlled trials and observational studies. J Shoulder Elbow Surg. (2016) 25(7):1195–203. 10.1016/j.jse.2016.01.018 27068381

[20] Lee YS Lin CC Huang CR Chen CN Liao WY. Operative treatment of midclavicular fractures in 62 elderly patients: knowles pin versus plate. Orthopedics. (2007) 30(11):959–64. 10.3928/01477447-20071101-13 18019991

[21] Calbiyik M Ipek D Taskoparan M. Prospective randomized study comparing results of fixation for clavicular shaft fractures with intramedullary nail or locking compression plate. Int Orthop. (2017) 41(1):173–9. 10.1007/s00264-016-3192-5 27138609

[22] Fuglesang HFS Flugsrud GB Randsborg PH Hammer OL Utvag SE. Five-year follow-up results of a randomized controlled study comparing intramedullary nailing with plate fixation of completely displaced midshaft fractures of the clavicle in adults. JB JS Open Access. (2018) 3(4):e0009. 10.2106/JBJS.OA.18.00009 30882049 PMC6400504

[23] King PR Ikram A Eken MM Lamberts RP. The effectiveness of a flexible locked intramedullary nail and an anatomically contoured locked plate to treat clavicular shaft fractures: a 1-year randomized control trial. J Bone Joint Surg Am. (2019) 101(7):628–34. 10.2106/JBJS.18.00660 30946197

[24] Andrade-Silva FB Kojima KE Joeris A Santos Silva J Mattar R Jr. Single, superiorly placed reconstruction plate compared with flexible intramedullary nailing for midshaft clavicular fractures: a prospective, randomized controlled trial. J Bone Joint Surg Am. (2015) 97(8):620–6. 10.2106/JBJS.N.00497 25878305

[25] Assobhi JE. Reconstruction plate versus minimal invasive retrograde titanium elastic nail fixation for displaced midclavicular fractures. J Orthop Traumatol. (2011) 12(4):185–92. 10.1007/s10195-011-0158-7 21948051 PMC3225608

[26] van der Meijden OA Houwert RM Hulsmans M Wijdicks FJ Dijkgraaf MG Meylaerts SA Operative treatment of dislocated midshaft clavicular fractures: plate or intramedullary nail fixation? A randomized controlled trial. J Bone Joint Surg Am. (2015) 97(8):613–9. 10.2106/JBJS.N.00449 25878304

[27] Ferran NA Hodgson P Vannet N Williams R Evans RO. Locked intramedullary fixation vs. plating for displaced and shortened mid-shaft clavicle fractures: a randomized clinical trial. J Shoulder Elbow Surg. (2010) 19(6):783–9. 10.1016/j.jse.2010.05.002 20713274

[28] Lee YS Huang HL Lo TY Hsieh YF Huang CR. Surgical treatment of midclavicular fractures: a prospective comparison of knowles pinning and plate fixation. Int Orthop. (2008) 32(4):541–5. 10.1007/s00264-007-0352-7 17364177 PMC2532272

[29] Narsaria N Singh AK Arun GR Seth RR. Surgical fixation of displaced midshaft clavicle fractures: elastic intramedullary nailing versus precontoured plating. J Orthop Traumatol. (2014) 15(3):165–71. 10.1007/s10195-014-0298-7 24859367 PMC4182648

[30] Yang H Wang LJ Bao QL. Intramedullary nailing versus plate fixation for midshaft clavicular fractures: a prospective randomized controlled trial. Int J Orthop. (2020) 41(3):180–3. 10.3969/j.issn.1673-7083.2020.03.012

[31] Bi SR Yang GP Zhou JG Liu ZY Liu SW Hu WQ Comparative study on the curative effect of elastic intramedullary needle and plate screw internal fixation in the treatment of adult Allman type I clavicle fractures. Med Innov China. (2022) 19(13):12–5. 10.3969/j.issn.1674-4985.2022.13.003

[32] Zehir S Zehir R Sahin E Calbiyik M. Comparison of novel intramedullary nailing with mini-invasive plating in surgical fixation of displaced midshaft clavicle fractures. Arch Orthop Trauma Surg. (2015) 135(3):339–44. 10.1007/s00402-014-2142-1 25552396

[33] Long Y Wei RJ Wei JH Liu Q Qin J Qin B. Clinical curative effect of elastic intramedullary nail combined with clavicle fixation band and anatomical titanium plate in the treatment of patients with middle clavicle fracture. J Trauma Surg. (2021) 23(12):940–3. 10.7759/cureus.17972

[34] Fridberg M Ban I Issa Z Krasheninnikoff M Troelsen A. Locking plate osteosynthesis of clavicle fractures: complication and reoperation rates in one hundred and five consecutive cases. Int Orthop. (2013) 37(4):689–92. 10.1007/s00264-013-1793-9 23377107 PMC3609968

[35] Houwert RM Wijdicks FJ Steins Bisschop C Verleisdonk EJ Kruyt M. Plate fixation versus intramedullary fixation for displaced mid-shaft clavicle fractures: a systematic review. Int Orthop. (2012) 36(3):579–85. 10.1007/s00264-011-1422-4 22146919 PMC3291769

[36] Barlow T Beazley J Barlow D. A systematic review of plate versus intramedullary fixation in the treatment of midshaft clavicle fractures. Scott Med J. (2013) 58(3):163–7. 10.1177/0036933013496960 23960055

[37] Duan X Zhong G Cen S Huang F Xiang Z. Plating versus intramedullary pin or conservative treatment for midshaft fracture of clavicle: a meta-analysis of randomized controlled trials. J Shoulder Elbow Surg. (2011) 20(6):1008–15. 10.1016/j.jse.2011.01.018 21481613

[38] Zhang B Zhu Y Zhang F Chen W Tian Y Zhang Y. Meta-analysis of plate fixation versus intramedullary fixation for the treatment of mid-shaft clavicle fractures. Scand J Trauma Resusc Emerg Med. (2015) 23:27. 10.1186/s13049-015-0108-0 25886940 PMC4372272

[39] Zhu Y Tian Y Dong T Chen W Zhang F Zhang Y. Management of the mid-shaft clavicle fractures using plate fixation versus intramedullary fixation: an updated meta-analysis. Int Orthop. (2015) 39(2):319–28. 10.1007/s00264-014-2655-9 25662762

[40] Chen YF Wei HF Zhang C Zeng BF Zhang CQ Xue JF Retrospective comparison of titanium elastic nail (TEN) and reconstruction plate repair of displaced midshaft clavicular fractures. J Shoulder Elbow Surg. (2012) 21(4):495–501. 10.1016/j.jse.2011.03.007 21641826

[41] Wijdicks FJ Houwert M Dijkgraaf M de Lange D Oosterhuis K Clevers G Complications after plate fixation and elastic stable intramedullary nailing of dislocated midshaft clavicle fractures: a retrospective comparison. Int Orthop. (2012) 36(10):2139–45. 10.1007/s00264-012-1615-5 22847116 PMC3460104

[42] Drosdowech DS Manwell SE Ferreira LM Goel DP Faber KJ Johnson JA. Biomechanical analysis of fixation of middle third fractures of the clavicle. J Orthop Trauma. (2011) 25(1):39–43. 10.1097/BOT.0b013e3181d8893a 21085028

